# Association Between Changes in BLyS Levels and the Composition of B and T Cell Compartments in Patients With Refractory Systemic Lupus Erythematosus Treated With Belimumab

**DOI:** 10.3389/fphar.2019.00433

**Published:** 2019-04-25

**Authors:** Francesca Regola, Silvia Piantoni, Torsten Lowin, Silvia Archetti, Rossella Reggia, Rajesh Kumar, Franco Franceschini, Paolo Airò, Angela Tincani, Laura Andreoli, Georg Pongratz

**Affiliations:** ^1^Department of Clinical and Experimental Sciences, University of Brescia, Brescia, Italy; ^2^Rheumatology and Clinical Immunology Unit, ASST-Spedali Civili of Brescia, Brescia, Italy; ^3^Department of Rheumatology and Hiller Research Centre for Rheumatology, University Hospital Düsseldorf, Düsseldorf, Germany

**Keywords:** B lymphocyte, T lymphocyte, BLyS levels, belimumab, systemic lupus erythematosus

## Abstract

**Introduction:** Belimumab is a monoclonal antibody against soluble BLyS used for treatment of refractory Systemic Lupus Erythematosus (SLE). Although B cells are the main target of this therapy, a BLyS-dependent T cell activation pathway has also been demonstrated. The aim of the study is to analyze B and T cells phenotype modifications in a cohort of SLE patients treated with belimumab in correlation with serum BLyS levels.

**Materials and Methods:** Fourteen SLE patients were enrolled in the study. Lymphocyte immunophenotyping by flow cytometry and determination of serum BLyS levels by high sensitivity ELISA were performed before the first infusion of belimumab, after 6 and 12 months of treatment. Sex and age-matched healthy controls were enrolled for the comparisons.

**Results:** Baseline number of total B cells, especially switched memory B cells, were lower in SLE patients compared to control subjects. After 6 months of treatment, the total number of B cells, particularly, naive and transitional B cells, was significantly reduced in correlation with the reduction of BLyS levels. No significant association was found between baseline counts of B cells and reduction of SLEDAI-2K over time. In terms of response prediction, a significant association between SLEDAI-2K improvement at 12 months and the decrease of total number of B cells within the first 6 months of therapy was observed. Concerning the T cell compartment, the baseline percentage number of CD8+ effector memory was associated with SLEDAI-2K at baseline and with its improvement after 12 months of therapy. Furthermore, T cell lymphopenia and low number of circulating recent thymic emigrants were also observed compared to control subjects measured at baseline.

**Discussion:** The effects of belimumab on B cell subpopulations could be explained by the direct blockage of soluble BLyS, while the mild effects on T cells might be explained indirectly by the reduction of disease activity by means of therapy. B cell immunophenotyping during belimumab might be useful for monitoring the response to treatment.

## Introduction

Systemic lupus erythematosus (SLE) is a systemic autoimmune disease characterized by the production of several autoantibodies. Both innate and adaptive immunity are involved in the pathogenesis of the disease, but recent evidences showed that adaptive immunity has a central role (Mak and Kow, 2014). An unbalance between effector and regulatory T cells (TREG), as well as the involvement of particular subsets of B cells, are described in the development of the disease (Tsokos et al., 2016; Piantoni et al., 2018). SLE patients have a severe defect in the B cell tolerance check, resulting in high numbers of autoreactive mature naïve B cells, which subsequently give rise to autoantibody producing plasma cells (Vossenkämper et al., 2011). The defect most likely occurs at the transitional stage between new bone marrow emigrants and mature naïve B cells in the periphery; hence, an expansion of transitional B cells may be detected in peripheral blood of SLE patients (Vossenkämper et al., 2011).

The mechanisms of selection of transitional B cells in humans are not yet fully understood (Sims et al., 2005); however, a crucial signal for the B cell development through this stage is thought to be delivered by B lymphocyte stimulator (BLyS, also known as BAFF), a member of the tumor necrosis factor superfamily (Mackay et al., 2010).

Belimumab is a human monoclonal antibody against soluble BLyS used in the treatment of SLE. In fact, BLyS overexpression leads to a lupus-like syndrome in mice, and its inhibition delays lupus onset in mouse models of spontaneous SLE (Boneparth and Davidson, 2012). Moreover, BLyS was found in high concentration in sera of SLE patients (Stohl et al., 2003).

A clinical trial study on 13 SLE patients treated with various dosages of belimumab demonstrated a selective depletion of naïve and transitional B cell subpopulations; in contrast, memory B cells and plasma cells were less susceptible to selective BLyS inhibition (Jacobi et al., 2010). Nevertheless, further studies are needed to confirm these findings.

Moreover, the presence of BLyS receptor 3 (also known as BAFF-R) on T cells and the role of a BLyS-dependent T cell activation pathway have been well demonstrated (Ng et al., 2004). Therefore, it may be hypothesized that belimumab could exert its action also through an effect on T cells. However, not much information is available on the effects of belimumab on circulating T cells (Jacobi et al., 2010).

The aim of this study was therefore to analyze the B and T cell phenotype of circulating cells in a cohort of SLE patients treated with belimumab and to correlate them with serum BLyS levels.

## Materials and Methods

### Patients

Fourteen consecutive patients with SLE, classified according to the revised American College of Rheumatology (ACR) criteria (Hochberg, 1997), and treated with belimumab according to common clinical practice were enrolled in this study. A written informed consent was obtained from all the patients. Their main clinical, laboratory and demographic features, obtained from clinical charts, are presented in Table 1. All patients participated in a previous study on T cell subsets characterization (Piantoni et al., 2018). SLE Disease Activity Index 2000 (SLEDAI 2K)-score was used to determine disease activity: a score ≥6 indicates high disease activity (Romero-Diaz et al., 2011). SLE responder index (SRI) index was used to evaluate the response after 12 months of treatment, as a reduction of ≥4 points in SLEDAI-2K, no new BILAG A or >1 new BILAG B, and no deterioration in PGA by ≥30 mm (Furie et al., 2009). Fourteen sex and age-matched healthy controls were enrolled for the comparisons. The study was approved by the local institutional ethical committee (approval number 2793) and conducted in accordance with the Declaration of Helsinki.

**Table 1:** Demographic, clinical and laboratory features of SLE patients at baseline.

**Demographic features**	
Women	11 (79%)
Age, years	38 (31–49)
Disease duration, years	13 (8–23)
Caucasian ethnicity	14 (100%)
**SLE manifestations *n* (%)**	
Cutaneous manifestations (malar rash and/or discoid rash, oral ulcers)	7 (50%)
Articular involvement (arthritis/Jaccoud’s arthropathy)	10 (71%)
Renal involvement	9 (64%)
Hematological involvement	7 (50%)
NPSLE	0
Serositis (pulmonary/pericardic effusion)	3 (21%)
Antiphospholipid Syndrome	3 (21%)
SLEDAI 2K-score	6 (3–11)
SDI	1 (0–2)
PGA	2 (1–2)
**Laboratory parameters**	
Reduced serum levels of C3 and/or C4	11 (79%)
Anti-dsDNA positivity	13 (93%)
aPL positivity ^∗^	5 (37%)
**Treatment**	
Steroids (prednisone)	13 (93%)
Dosage of prednisone (mg/week)	38 (25–135)
Use of hydroxychloroquine at 5 mg/Kg/day	11 (79%)
Use of immunosuppressive drugs ^∗∗^	11 (79%)


*Data are expressed as median (10th–90th percentile), if not otherwise specified. NPSLE, neuropsychiatric systemic lupus erythematosus; SLEDAI-2K score, Systemic Lupus Erythematosus Disease Activity Index 2000; SDI, Systemic Lupus International Collaborating Clinics/American College of Rheumatology Damage Index; PGA, Physician Global Assessment; Anti-dsDNA, anti-double-stranded DNA autoantibody; aCL, anticardiolipin antibodies; Ig, immunoglobulin; Anti-b2GPI, anti-beta2-glycoprotein I antibodies; LA, lupus anticoagulant; nv, normal values. ^∗^1 patient with triple, 2 patients with double, and 3 patients with single positivity for aPL. ^∗∗^3 patients were on treatment with mycophenolate mofetil, 2 patients with methotrexate, 2 with azathioprine, 2 with cyclosporine, 1 with leflunomide, and 1 one with both methotrexate and cyclosporine. The immunosuppressive therapy remained the same at T6 and at T12.*

### Methods

#### Flow Cytometry

**FIGURE 1:**
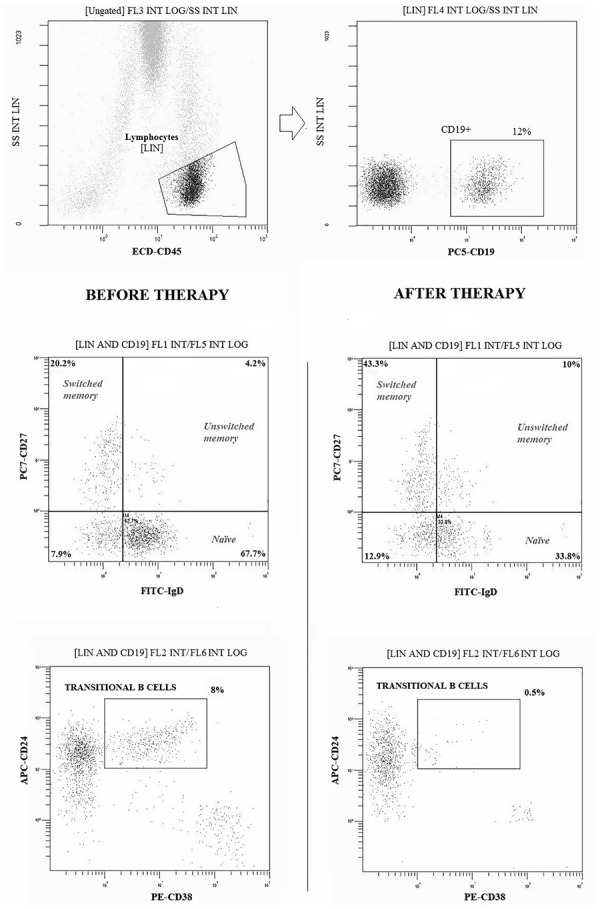
Gating strategies and dot-plot analysis of B (CD19+) cell subpopulations in a representative patient before and after 12 months of therapy with belimumab. Naïve: CD27-IgD+; Switched Memory: CD27+IgD-; Unswitched Memory: CD27+IgD +; Transitional: CD24 high CD38 high. At least 20.000 events were analyzed for each sample.

Peripheral blood samples from all patients and controls were obtained at the start of the study (baseline = T0) and every 6 months of treatment (6 months = T6 and 12 months = T12). To identify T cell surface markers, 100 μl of whole blood were stained for 30 min at 4°C using monoclonal antibodies conjugated with fluorochromes (Beckman Coulter) and read by flow cytometry (Cytomics NAVIOS, Beckman Coulter Inc., Fullerton, CA, United States), as previously described (Tsokos et al., 2016). Moreover, FITC-CD31, PE-CD25, ECD-CD45RA, PC5-CD4, and PC7-CD127 were used to identify T-cells recent thymic emigrants (RTE; CD31+CD45RA+) among Treg (CD4+CD25+) and other CD4+ T-cell subsets. B-cell subpopulations were evaluated with FITC-IgD, PE-CD38, ECD-CD45, PC5-CD19, PC7-CD27, and APC-CD24, as shown in Figure 1. Absolute cells counts was determined by single platform analysis using Flow-Count beads (Beckman Coulter).

#### ELISA

Serum samples of 10 patients collected every 6 months (T0, T6, and T12) were measured by R&D BAFF ELISA human Duo Set for the determination of BLyS levels, according to manufacturer’s guidelines.

### Statistical Analysis

Comparisons between healthy controls and patients were made with Mann–Whitney test. The variations between baseline and different time points were evaluated using Wilcoxon signed-rank test or Friedmann ANOVA on ranks. Pearson correlation/linear regression or logarithmic regression were used to evaluate the association between quantitative variables. All relevant associations were confirmed using linear modeling and controlling for lymphocyte numbers throughout. Adjustment for inter-individual patient effects was done by including a random intercept in the linear regression model. Statistical analysis was performed by using the software package StatView (SAS Institute Inc., Cary, NC, United States), SPSS (version 25.0, SPSS, Chicago, IL, United States) and R software package version 3.5.2. [R Core Team (2018), R Foundation for Statistical Computing, Vienna, Austria]. A *p*-value (*p*) ≤ 0.050 was considered as statistically significant.

## Results

### Characterization of Differences in Baseline Composition of B and T Cell Compartments Between SLE Patients and Healthy Controls

A lower absolute count of total circulating CD19+ B cells (*p* = 0.050; Table 2), and in particular, a lower number of CD19+ switched memory cells was observed in patients with SLE at baseline, as compared to healthy controls (*p* = 0.01, Table 2). As previously reported (Piantoni et al., 2018), T cell lymphopenia was observed in patients with SLE, with increased percentages of effector cells (data not shown). Moreover, patients with SLE had lower absolute number of circulating RTE (*p* = 0.03).

**Table 2:** Comparisons among the number of B- and T-cell subsets (expressed as percentage and absolute number) between patients and healthy controls, and between patients before and after 6 and 12 months of treatment with belimumab.

	HC (*n* = 14)	T0 (*n* = 14)	T6 (*n* = 14)	T12 (*n* = 13)	p HC vs. patients at T0 (*n* = 14)	PT0 vs. T6 (*n* = 14)	p T0 vs. T12 (*n* = 13)	p T6 vs. T12 (*n* = 13)
**B Cells**								
CD19+ (% lymphocytes)	8 (6-9)	8 (3-22)	5 (2-7)	3 (1-5)	0.70	**0.02**	**0.0022**	**0.02**
CD19+ (cell/μl)	153 (120-229)	82 (15-361)	23 (11-57)	19 (6-55)	**0.050**	0.0088	0.0047	0.72
CD19+ SW (% CD19+)	17 (12-24)	16 (5-39)	35 (15-56)	44 (16-61)	0.80	0.0037	0.0046	0.15
CD19+ SW (cell/μl)	28 (19-48)	11 (5-29)	6 (2-20)	4 (2-25)	**0.01**	0.24	0.53	0.79
CD19+ UNSW (% CD19+)	2 (0-8)	5 (0-13)	6 (2-20)	7 (3-12)	0.20	0.27	0.86	0.19
CD19+ UNSW (cell/μl)	3 (2-12)	2 (1-9)	2 (1-6)	1 (0-4)	0.50	0.17	**0.050**	0.24
CD19+ naïve (% CD19+)	62 (40-71)	45 (15-82)	21 (8-43)	19 (8-35)	0.20	**0.0019**	**0.0019**	0.15
CD19+ naïve (cell/μl)	97 (40-155)	36 (4-235)	4 (2-13)	5 (1-10)	0.20	**0.0024**	**0.0029**	0.37
TRANSITIONAL (% CD19+)	1 (0-3)	0.6 (0-8)	0.5 (0-4)	0.5 (0-6)	0.60	0.38	0.63	0.28
TRANSITIONAL (cell/μl)	1 (0-4)	1 (0-9)	0 (0-1)	0.2 (0-1)	0.80	**0.03**	**0.03**	0.33
**T cells**						
CD4+ (% lymphocytes)	49 (40-65)	43 (30-64)	47 (30-55)	46 (34-58)	0.20	0.50	0.10	0.50
CD4+ (cell/μl)	1131 (716-1370)	365 (97-1002)	326 (170-693)	317 (99-592)	**0.0004**	0.07	0.90	0.50
CD4+ RTE (% CD4+)	18 (9-25)	20 (14-38)	17 (3-35)	10 (3-27)	0.20	0.30	**0.01**	0.07
CD4+ RTE (cell/μl)	240 (70-286)	95 (31-177)	68 (6-106)	30 (18-74)	**0.03**	0.09	0.10	0.30
CD8+ (% lymphocytes)	25 (20-30)	27 (19-43)	32 (18-40)	31 (20-41)	0.40	0.90	0.10	0.70
CD8+ (cell/μl)	516 (241-733)	340 (85-501)	258 (67-414)	254 (86-410)	**0.01**	**0.050**	0.80	0.70
CD8+ EM (% CD8+)	32 (13-45)	26 (15-37)	22 (14-37)	26 (9-51)	0.30	0.60	0.60	0.10
CD8+ EM (cell/μl)	133 (14-286)	61 (24-118)	53 (10-110)	54 (16-174)	0.09	**0.04**	0.50	**0.050**


*Data are expressed as median (10th–90th percentile). HC, Healthy Controls; SW, Switched B-cells; UNSW, Unswitched B-cells; EM, Effector memory T-cells; RTE, Recent Thymic Emigrants T-cells. Mann–Whitney test or Wilcoxon signed-rank test was used for the comparisons, when appropriate. In bold *p* ≤ 0.050.*

### Clinical and Laboratory Features of SLE Patients Treated With Belimumab

One patient moved to another center and was lost during follow-up after 7 months of treatment. During belimumab treatment there was a significant reduction in SLEDAI 2K activity index (Table 3). At baseline, eight patients had high disease activity. At T6, 11 patients reached low disease activity, which was maintained at T12 in 10 cases. On the other hand, 2 out of 3 patients with SLEDAI 2K-score ≥6 at T6 reached low disease activity at T12. Prednisone dosage was progressively reduced (Table 3). Other therapies were unchanged. Serum levels of BLyS significantly decreased over time (Table 3). T0-T12 SRI index showed a response to the treatment in 69% of our patients.

**Table 3:** Comparisons of the clinical and laboratory features of SLE patients at different time points.

	T0 (*n* = 14)	T6 (*n* = 14)	T12 (*n* = 13)	Friedman’s ANOVA on ranks (*p*-value)
**Disease activity**				
SLEDAI 2K-score	7.0 (3.7)	3.7 (2.2)	3.5 (1.2)	**<0.001**
**Laboratory parameters**				
Serum levels of C3 (mg/dl) (nv = 80–160)	74.4 (20.6)	73.1 (22.2)	70.4 (22.2)	0.417
Serum levels of C4 (mg/dl) (nv = 10–40)	12.1 (6.2)	13.3 (5.9)	12.3 (6.3)	0.804
Serum levels of anti-dsDNA (UI/ml) (nv < 7)	93.4 (186.7)	90.6 (127.7)	62.0 (73.9)	0.629
BLyS levels (pg/ml)^∗^	572.7 (571.0)	396.7 (344.9)	387.0 (335.5)	**0.045**
**Treatment**				
Dosage of prednisone (mg/week)	56.9 (50)	32.2 (19.2)	25.2 (15.6)	**0.011**


*Data are expressed as mean (standard deviation). SLEDAI-2K score, Systemic Lupus Erythematosus Disease Activity Index 2000; Anti-dsDNA, anti-double-stranded DNA autoantibody; BLyS, B lymphocyte stimulator; nv, normal values; ns, not significant. Friedman’s ANOVA on ranks was used for the comparisons. In bold *p* ≤ 0.050. ^∗^BLyS levels were tested in only 10 patients.*

### Analysis of Changes in the B Cell Compartment in SLE Patients During Follow-Up While on Belimumab Treatment

As shown in Table 2, after treatment with belimumab CD19+ B lymphocytes decreased in patients with SLE, both in percentages (T0 vs. T6: *p* = 0.02; T6 vs. T12: *p* = 0.02; T0 vs. T12: *p* = 0.002) and absolute numbers (T0 vs. T6: *p* = 0.009; T0 vs. T12: *p* = 0.005).

In particular, there was a decrease of naïve B cells, in percentages (T0 vs. T6: *p* = 0.002; T0 vs. T12: *p* = 0.002) and absolute numbers (T0 vs. T6: *p* = 0.002; T0 vs. T12: *p* = 0.003), and of transitional B cells absolute number (T0 vs. T6: *p* = 0.03; T0 vs. T12: *p* = 0.03). The percentage of switched memory B cells increased (T0 vs. T6: *p* = 0.004; T0 vs. T12: *p* = 0.005), but their absolute number did not change. A linear model controlling for lymphocyte numbers confirmed these results (data not shown). A reduction of unswitched memory B cells after 12 month of therapy was shown in initial analysis (T0 vs. T12: *p* = 0.05), but the effect was not robust after controlling for lymphocytes.

### Analysis of Changes in the T Cell Compartment in SLE Patients During Follow-Up While on Belimumab Treatment

Comparing distributions of memory, effector and regulatory T cell subsets before and after therapy with belimumab, we did not observe any differences, except for a reduction of the absolute number of CD8+ cells at T6 only (*p* = 0.050), especially CD8+ EM cells (T0 vs. T6: *p* = 0.04; T6 vs. T12: *p* = 0.050). Furthermore, a reduced percentage of total RTE cells in the CD4+ T cell compartment was noticed following treatment for 12 months (T0 vs. T12: *p* = 0.01; Table 2). After controlling for lymphocytes using linear mixed modeling, we confirmed a reduced percentage of CD4+ RTE and found an additional reduction of absolute number of CD4+ RTE cells after 12 months of therapy (*R*^2^ = 0.478, estimate: -50.98, CI: -90.92 --11.05, *p* = 0.02). Furthermore, we find also a reduction of the absolute number of naïve CD4+ T cells after 12 months of therapy (*R*^2^ = 0.640, estimate: -36.84, CI: -71.40 --2.28, *p* = 0.048).

### Correlation Between Changes in B and T Cell Compartments and BLyS Levels

The relative change of BLyS levels at 6 and 12 months from baseline showed linear correlations with the percentage of naïve B cells (Pearson correlation = 0.645, *p* = 0.044 and 0.639, *p* = 0.002, respectively) and transitional B cells (Pearson correlation = 0.768, *p* = 0.009 and 0.623, *p* = 0.055, respectively). No correlations were found within the T cell compartment. After the correction for lymphocyte counts, relative change of BLyS was associated as an independent factor with a reduction in the number of naïve B cells (*R*^2^ = 0.605, estimate: 0.02, CI = 0–0.03, *p* = 0.023).

### Correlation Between Changes in the B and T Cell Compartment and Clinical Parameters During Follow-Up

No significant association was found between the baseline values of B cell numbers and the reduction of SLEDAI-2K over time (data not shown). However, at T12 the only 2 patients with high disease activity had higher percentages of transitional B cells and unswitched memory cells than 7 patients in which an initially high SLEDAI-2K decreased below 6 [9% (7–11) vs. 1% (0–4) and 29% (17–41) vs. 6% (4–8), respectively].

**FIGURE 2:**
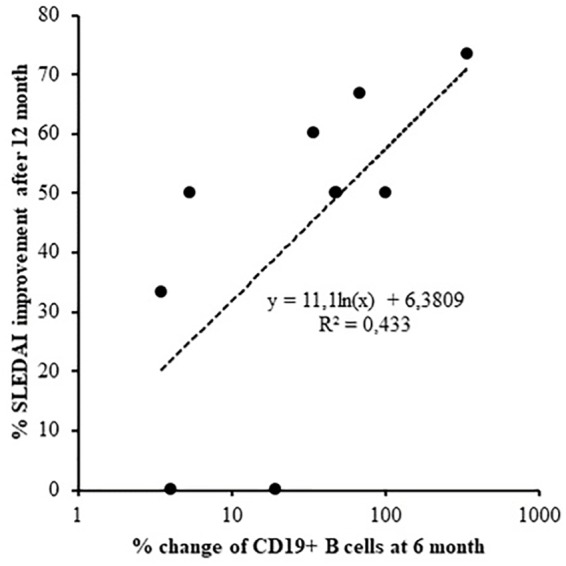
Logarithmic regression between SLEDAI-2K change after 12 months of treatment and the reduction of CD19 cells after 6 months (Log regression, *r* = 0.658, *p* = 0.039).

In terms of response prediction, percent of SLEDAI-2K improvement at 12 months was associated with a decrease in total number of B cells within the first 6 months of therapy (Log regression, *r* = 0.658, *p* = 0.039; Figure 2).

**FIGURE 3:**
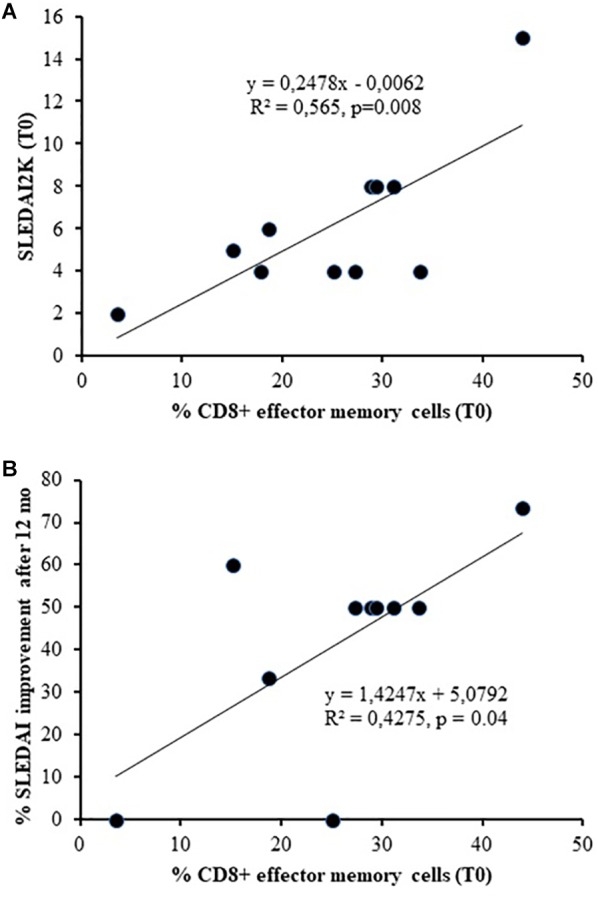
Association between the baseline percentage number of CD8+ Effector Memory and SLEDAI-2K at T0 **(A)** (linear regression, *r* = 0.752, *p* = 0.008) and its improvement after 12 months of therapy **(B)** (linear regression, *r* = 0.654, *p* = 0.04).

Concerning T cell compartment, the baseline percentage of CD8+ EM was associated with SLEDAI-2K at T0 (linear regression, *r* = 0.752, *p* = 0.008) and with its improvement after 12 months of therapy (linear regression, *r* = 0.654, *p* = 0.04, Figure 3).

Performing logistic regression on SRI response showed no significant associations (data not showed).

## Discussion

Since lymphopenia is a common clinical manifestation and one of the classification criteria for SLE (Hochberg, 1997), the low absolute number of B and T cells observed in our patients at baseline were expected. The reduced number of RTE confirms data suggesting a reduced thymic output in patients with active SLE (Vieira et al., 2008), one of the possible causes of T cell lymphopenia.

After treatment with belimumab, we observed a reduction of B cells and of their naïve and transitional subpopulations. Our results confirm, in a real-life scenario, a previous study on 13 patients participating in a clinical trial and treated with various dosages of belimumab (in most cases, lower than in common clinical practice) (Jacobi et al., 2010). In this study the depletion of naïve and transitional B cells was significant after 3 months of therapy, whereas, the reduction of unswitched memory B cells became evident only after 12 months, similarly to our observation. Moreover, we confirm that the inhibition of BLyS did not influence the switched memory cell compartment (Jacobi et al., 2010). A short time initial expansion of memory B cells, which later returned to baseline levels, was demonstrated by more recent studies, without significant variations in anti vaccine antibody titers during time (Stohl et al., 2003; Ramsköld et al., 2018). It may be secondary to their release from germinal centers or to inhibition of their return to lymphoid tissues, or it may be consequent to promotion of differentiation of naïve to memory B cells (Stohl et al., 2003).

B cell survival is regulated by the crosstalk between B cell receptor (BCR) and BLyS-R pathways: BCR cross-link on immature B cells may induce apoptosis, which can be prevented by BLyS, whereas in mature B cells it can stimulate cell activation and growth (Mackay et al., 2010). The selective effects of belimumab on circulating B cells may therefore be explained by the differential expression of various BLyS receptors in different phases of B cell maturation: BLyS-R has a strong selectivity for BLyS and is preferentially expressed on naïve and transitional B cells, whereas BLyS inhibition by belimumab cannot block signaling mediated by APRIL through other receptors such as TACI and BCMA, which are expressed in other phases of B cell ontogeny (Mackay et al., 2010). The correlation between the relative change of BLyS levels and the modifications of the naïve and transitional B cell number in our cohort confirms this evidence. This correlation was shown to be weak as demonstrated by the application of the linear model corrected for lymphocyte counts. This might indicate that the main effect of belimumab on subpopulations could be not via serum BLyS (which we measured) but may be via blocking membrane BLyS. However, the effect on unswitched memory cells is delayed and might be explained by the reduced production of marginal-zone B cells that are expected to be BLyS-dependent (Jacobi et al., 2010).

Since survival of autoreactive B cells depends more on the interaction between BLyS and its receptors than on the stimulation of BCR, which is downregulated in these cells, differently from their non-reactive counterparts, it may be hypothesized that belimumab can promote the deletion of autoreactive B cells, especially in their first stages of maturation (Liu and Davidson, 2011). However, this has not yet been demonstrated in SLE patients, and the effects on autoreactive plasma cells and autoantibodies are modest (Jacobi et al., 2010). The clinical efficacy of belimumab might be, at least in part, independent of autoantibody depletion.

Similarly, no direct correlation of B cell number variations with clinical features or anti-dsDNA was found in our cohort. Only an association between SLEDAI-2K percentage improvement after 12 months of therapy and the decrease of total number of B cells within the first 6 months was found, showing their possible role as predictor of response.

Moreover, patients with high disease activity after 12 months of treatment had higher percentages of transitional and unswitched B cells at T12, suggesting that the number of circulating B cells belonging to these subsets might also be used as a marker of response to belimumab. This was confirmed by a recent study which suggested that evaluation of B cell counts might be useful at the beginning of belimumab treatment and that high baseline B cell counts predicted no response to the treatment (Ramsköld et al., 2018). Future studies are needed to evaluate the possible mechanisms underlining the failure of this therapeutic approach: among other possible explanations, involvement of BLyS-independent mechanisms of B cell survival, the presence of natural BLyS-neutralizing autoantibodies, or the rise of anti-drug antibodies would be interesting to look for.

The possible effect of belimumab on T cells was hypothesized considering that *in vitro* studies of human T cells showed that BLyS can provide a complete costimulatory signal together with anti-T cell receptor (TCR) stimulation (Huard et al., 2004). In the above mentioned study by Jacobi et al. (2010), no variation of T cell counts was observed, but effector memory T cells were not evaluated. Indeed, a reduction of CD8+ EM was the only variation among T cell subpopulations observed in our cohort. Their baseline percentage number was associated with SLEDAI-2K at T0 and with its improvement after 12 months of therapy, suggesting the possible role of CD8+ EM as a useful marker of response to treatment. However, it cannot be excluded that the variation of their number can indirectly be explained by the reduction of disease activity obtained through the therapy. In fact, effector T cells are particularly expanded in patients with high disease activity (Piantoni et al., 2018). *Vice versa*, the lack of RTE defect correction observed in SLE patients treated with belimumab suggests that the reduced thymic output cannot be corrected by reducing disease activity through an action on BLyS.

In conclusion, our results confirm the effects of belimumab on B cell subpopulations. These can be directly explained by the blockage of soluble BLyS interaction with BLyS-R. On the other hand, the effects on the composition of the T cell compartment are mild, which is not totally unexpected, since the intensity of BLyS-R expression by peripheral T cells is low (Ng et al., 2004). The principal limits of this study include the small sample size, presence of concomitant immunosuppressive therapies and low absolute cell number at baseline. Nevertheless, future studies on larger numbers of patients are needed to evaluate whether changes in B cell subsets after therapy initiation could be considered as marker of response to treatment with belimumab in SLE patients.

## Ethics Statement

The study was approved by the institutional ethical committee of Brescia (approval number 2793) and conducted in accordance with the Declaration of Helsinki.

## Author Contributions

FR, SA, SP, and TL designed, set up, and carried out the experiments. GP, FR, RK, and SP analyzed the data. LA, RR, FF, PA, and AT performed the clinical evaluation of the patients and the collection of the samples. LA, SP, AT, GP, FF, and PA designed and supported the study. All authors checked the final version of the manuscript.

## Conflict of Interest Statement

LA, FF, and AT received speaking fees from Glaxo Smith Kline. The remaining authors declare that the research was conducted in the absence of any commercial or financial relationships that could be construed as a potential conflict of interest.
